# Growth differentiation factor-15 and white matter hyperintensities in cognitive impairment and dementia

**DOI:** 10.1097/MD.0000000000004566

**Published:** 2016-08-19

**Authors:** Yuek Ling Chai, Saima Hilal, Jenny P.C. Chong, Yan Xia Ng, Oi Wah Liew, Xin Xu, Mohammad Kamran Ikram, Narayanaswamy Venketasubramanian, A. Mark Richards, Mitchell K.P. Lai, Christopher P. Chen

**Affiliations:** a Department of Pharmacology, Yong Loo Lin School of Medicine, National University of Singapore; b Memory Aging and Cognition Centre, National University Health System; c Cardiovascular Research Institute, National University Heart Centre, Singapore; d Departments of Neurology and Epidemiology, Erasmus University Medical Center, Rotterdam, the Netherlands; e Raffles Neuroscience Centre, Raffles Hospital, Singapore.

**Keywords:** biomarker, cerebrovascular disease, cognitive impairment, dementia, growth differentiation factor-15, white matter hyperintensities

## Abstract

Vascular pathology plays an important role in the development of cognitive decline and dementia. In this context, growth differentiation factor-15 (GDF-15) has been suggested to be a biomarker due to its regulatory roles in inflammatory and trophic responses during tissue injury. However, limited data exist on the associations of GDF-15 with either cerebrovascular disease (CeVD) burden or the spectrum of cognitive impairment. Therefore, we aimed to study peripheral levels of GDF-15 incognitive impairment no dementia (CIND) or Alzheimer disease (AD) subjects assessed for CeVD using a case–control cohort design, with cases recruited from memory clinics and controls from memory clinics and the community. All subjects underwent detailed neuropsychological assessment, 3-Tesla magnetic resonance imaging, and venous blood draw. Subjects were classified as CIND or AD based on clinical criteria, while significant CeVD was defined as the presence of cortical infarcts and/or 2 lacunes or more, and/or confluent white matter hyperintensities (WMHs) in 2 or more brain regions. A total of 324 subjects were included in the study, of whom 80 had no cognitive impairment, 144 CIND and 100with AD. Higher GDF-15 levels were significantly associated with disease groups, especially in the presence of CeVD, namely, CIND with CeVD (odds ratios [OR]: 7.21; 95% confidence interval [CI]: 2.14–24.27) and AD with CeVD (OR: 21.87; 95% CI: 2.01–237.43). Among the different CeVD markers, only WMH was associated with higher GDF-15 levels (OR: 3.97; 95% CI: 1.79–8.83). The associations between GDF-15 and cognitive impairment as well as with WMH remained significant after excluding subjects with cardiovascular diseases. In conclusion, we showed that increased GDF-15 may be a biomarker for CIND and AD in subjects with WMH.

## Abstract 2

Supplemental Digital Content is available in the text

## 1 Introduction

There is accumulating evidence showing the involvement of cerebrovascular disease (CeVD) in the development of Alzheimer disease (AD). While amyloid plaques and neurofibrillary tangles are recognized as the pathological hallmarks of AD, markers of CeVD, such as white matter hyperintensities (WMHs), lacunar infarcts, and cerebral microbleeds, have been reported in AD brains.^[1]^ Moreover, both CeVD and AD pathology have common risk factors, such as age, hypertension, diabetes, stroke, smoking, and cardiovascular diseases, suggesting a pathogenic role of vascular disease in AD.^[2]^ Furthermore, presence of CeVD may act synergistically or additively with AD in contributing to dementia severity.^[3–5]^ In this context, vascular markers such as growth differentiation factor-15 (GDF-15) have recently gained research interest as potential biomarkers for cognitive decline.^[6]^

GDF-15, also known as macrophage inhibitory cytokine-1 or NSAID-activated gene, is an anti-inflammatory, proapoptotic, stress response cytokine belonging to the transforming growth factor beta superfamily.^[7,8]^ It can be found in various tissues and organs, and is generally elevated in injury to brain, liver, or heart.^[9–11]^ GDF-15has also been shown *in vitro* to have protective and trophic functions for both neurons and cardiomyocytes.^[11–13]^ Clinical studies have reported associations between elevated blood GDF-15 levels and increased cardiovascular risk and mortality.^[14,15]^ However, given the increasing recognition of a “heart-brain connection,” where cardiovascular risk factors are related to cerebrovascular risk factors and their sequelae, including cognitive impairment,^[16]^ we hypothesized that GDF-15 may also be a marker for cognitive impairment via its association with CeVD. Indeed, GDF-15 has been found to predict for adverse outcomes after ischemic stroke.^[17,18]^ However, only 1 study to date has reported GDF-15 elevation as a predictor for worse cognitive performance in patients with mild cognitive impairment,^[6]^ and another study showed GDF-15 was associated with worse performance in a specific cognitive test (visual reproduction) and WMH in a nondemented cohort^[19]^ (see Jiang et al^[20]^ for a review). In this study, we aimed to investigate the association of GDF-15 with cognitive impairment no dementia (CIND) as well as AD. Given the potential involvement of GDF-15 in vascular diseases, the putative associations between GDF-15 and CeVD as well as other vascular risk factors were also explored.

## 2 Materials and methods

### 2.1 Study population

The present study adopts a case–control design. Cases (CIND and AD) with subjective complaints of memory loss and cognitive impairments on neuropsychological assessment were recruited from 2 study sites in Singapore (ie, the memory clinics from National University Hospital and Saint Luke's Hospital). Cognitively normal controls (no cognitive impairment, NCI) were recruited from both memory clinics and the community. Controls (from memory clinic and community) were defined as cognitively normal on objective neuropsychological assessment.^[21]^ Ethics approval for this study was obtained from National Healthcare Group Domain-Specific Review Board. The study was conducted in accordance with the Declaration of Helsinki. Written informed consent was obtained for all participants in their preferred language before study recruitment.

### 2.2 Examination procedures

All subjects underwent standard physical, clinical, blood tests, and neuropsychological assessments as well as neuroimaging scans at the National University of Singapore. The detailed study procedures have been described previously.^[21]^

### 2.3 Blood biomarkers measurement

Non-fasting blood was drawn from study participants into ethylenediaminetetraacetic acid tubes and processed by centrifugation at 2000×*g* for 10 minutes at 4°C, followed by extraction of the upper plasma layer and storage at −80°C until use. GDF-15 concentrations were measured by a quantitative sandwich immunoassay technique (Quantikine, Catalogue number DGD150, R&D Systems, Inc. Minneapolis, MN) in accordance to manufacturer's instructions. Plasma ethylenediaminetetraacetic acid samples were diluted 4-fold in assay diluent buffer before addition to the capture antibody-coated plate. Detection was achieved by the addition of the chromogenic substrate, tetramethylbenzidine, and color development was stopped after 30 minutes incubation by the addition of sulfuric acid. Absorbance was measured at 450 nm on a multimode microplate reader (Perkin Elmer, Waltham, MA) with background subtraction at 570 nm. A 7-calibrator standard curve ranging from 23.4 to 1500 pg/mL was generated for each assay and fitted to a 5-parameter logistic model with weighted R-squared correlation coefficient of more than 0.99. Sample concentrations read from the standard curve were multiplied by the dilution factor of 4 to obtain the actual GDF-15 circulating levels in plasma. Quality control (QC) samples from R&D Systems were included in each assay for which the in-house established mean concentrations of low, medium, and high QC samples based on 156 independent assays were 158 (standard deviation, SD = 8.39), 436 (SD = 24.5), and 832 (SD = 73.3) pg/mL, and interassay coefficient of variation (%CV) of 5.3%, 5.6%, and 8.8%, respectively.

GDF-15 plasma levels of the whole cohort were measured in duplicates over 12 independent assays. Results were accepted when at least 2 of 3 QCs fell within control limits of ± 2 standard deviations and none exceeding ± 3 standard deviations of the in-house established QC mean values. The mean values of the low, medium, and high QCs for the 12 assays were 157 pg/mL (interassay %CV = 5.46), 422 pg/mL (interassay %CV = 10.7), and 799 pg/mL (interassay %CV = 15.8%), respectively. Overall range of GDF-15 detection was 313 to 9053 pg/mL. For sample measurements, the mean intra-assay coefficient of variation was 1.95% (range = 0%–12.3%). The interassay coefficient of variation (n = 3 independent assays measured in duplicates) at 1124, 3310, and 6463 pg/mL were 7.49%, 10.3%, and 5.12%, respectively. All the blood samples were analyzed blinded to subject characteristics and clinical status.

### 2.4 Neuroimaging

Magnetic resonance imaging (MRI) scans were performed on a 3-Tesla Siemens Magnetom Trio Tim scanner, using a 32-channel head coil, at the Clinical Imaging Research Centre, National University of Singapore. Subjects with claustrophobia, contraindications for MRI, or those who were unable to tolerate the procedure were excluded. All MRIs were graded by 1 radiologist and 2 clinicians blinded to the neuropsychological and clinical data. The sequences included T1-weighted Magnetization Prepared Rapid Gradient Recalled Echo, Fluid Attenuated Inversion Recovery, T2-weighted, and Susceptibility Weighted Imaging sequences. Presence of lacunes and cortical infarcts were defined on Fluid Attenuated Inversion Recovery and T2 sequences using STRIVE criteria,^[22]^ whereas WMH were graded using the Age-Related White Matter Changes scale (ARWMC).^[23]^ Significant CeVD was defined as the presence of cortical strokes and/or 2 lacunes or more, and/or confluent WMH (ARWMC score ≥8) in 2 regions of the brain, as described previously.^[21]^

### 2.5 Neuropsychological assessment

Cognitive tests, which included the Mini-Mental State Examination, the Montreal Cognitive Assessment and a locally validated, detailed neuropsychological test battery,^[24]^ were administered to all subjects by trained research psychologists. The test battery assessed 7 cognitive domains, 5 of which were nonmemory domains. The nonmemory domains were Executive Function (using Frontal Assessment Battery^[25]^ and Maze Task^[26]^); Attention (using Digit Span, Visual Memory Span,^[27]^ and Auditory Detection^[28]^); Language (using Boston Naming Test^[29]^ and Verbal Fluency^[30]^); Visuomotor Speed (using Symbol Digit Modality Test^[31]^ and Digit Cancellation^[32]^) and Visuoconstruction (using Weschler Memory Scale-Revised Visual Reproduction Copy task,^[27]^ Clock Drawing,^[33]^ and Weschler Adult Intelligence Scale-Revised subtest of Block Design^[34]^). The memory domains assessed were Verbal Memory (using Word List Recall^[35]^ and Story Recall) and Visual Memory (using Picture Recall and Weschler Memory Scale-Revised Visual Reproduction^[27]^).

### 2.6 Diagnosis of cognitive impairment and dementia

Diagnoses of cognitive impairment and AD were made at weekly consensus meetings by study clinicians and neuropsychologists. CIND was determined by clinical judgment based on published guidelines,^[36]^ namely, impairment in at least one domain of the neuropsychological test battery without any significant dysfunction in activities of daily living. Participants were considered to have failed a test if they scored 1.5 SD (standard deviation) below education-adjusted cutoff values on each individual test. Failure in at least half of the tests in each domain was considered as impairment in that domain. The diagnosis of AD was based on the Diagnostic and Statistical Manual of Mental Disorders, 4^th^ edition criteria and the National Institute of Neurological and Communicative Disorders and Stroke and the Alzheimer Disease and Related Disorders Association (NINCDS-ADRDA) criteria.^[37]^ Because the present study focuses on GDF-15 as a potential biochemical marker of CeVD in AD, subjects diagnosed to have a primary vascular cause for dementia (eg, vascular dementia [VaD] diagnosed using the National Institute of Neurological Disorders and Stroke-Association Internationale pour la Recherché et l’ Enseignement en Neuroscience criteria^[38]^) were not included in analyses.

### 2.7 Other risk factors assessment

Risk factors, such as hypertension, hyperlipidemia, diabetes, smoking, and cardiovascular diseases were ascertained from clinical interview and medical records and classified as present or absent. Hypertension was defined as systolic blood pressure of 140 mm Hg or more and/or diastolic blood pressure 90 mm Hg or more, or use of antihypertensive medications. Diabetes mellitus was defined as glycated hemoglobin of 6.5% or more, or on medication. Hyperlipidemia is defined as total cholesterol levels of 4.14 mM or more, or on medication. Cardiovascular disease was classified as a previous history of atrial fibrillation, congestive heart failure, and myocardial infarction. Because anti-inflammatory medication including NSAIDs are known to affect GDF-15 expression,^[8]^ we also recorded the use of NSAIDS and other anti-inflammatories like aspirin or paracetamol. Education was categorized as low education (not exceeding elementary education) and higher education (higher than elementary education). Smoking was categorized as present (ever) and absent (never).

### 2.8 Statistical analysis

Statistical analyses were performed using Statistics software (version 21, IBM SPSS, Armonk, NY). Analyses of variance and chi-square tests were used to compare the characteristics of the cases and controls groups. Because GDF-15 was not normally distributed (Shapiro–Wilk test *P* < 0.001, skewness = 2.62, and kurtosis = 8.13), GDF-15 levels were categorized into tertiles and included as a determinant, whereas CIND and dementia were defined as outcomes. Binary logistic regression analysis with odds ratios (OR) and 95% confidence intervals (95% CI) were first computed for CIND and AD. Further regression analyses were performed for both CIND and AD stratified by significant CeVD on MRI. The models were adjusted for age, education, hypertension, diabetes, intake of anti-inflammatory medication, and cardiovascular disease as covariates, as some of these variables were not matched between groups, while others are known *a priori* to affect GDF-15 levels (see Table 1). In order to identify specific associations between CeVD markers and GDF-15, we further performed logistic regressions separately for WMH (ARWMC ≥ 8), presence of lacunes and cortical infarcts adjusting initially for age, gender, and subsequently for other risk factors, namely, hypertension, hyperlipidemia, smoking, intake of anti-inflammatory medication, cardiovascular diseases, and other MRI markers. *P* values < 0.05 were considered statistically significant.

**Table 1:**
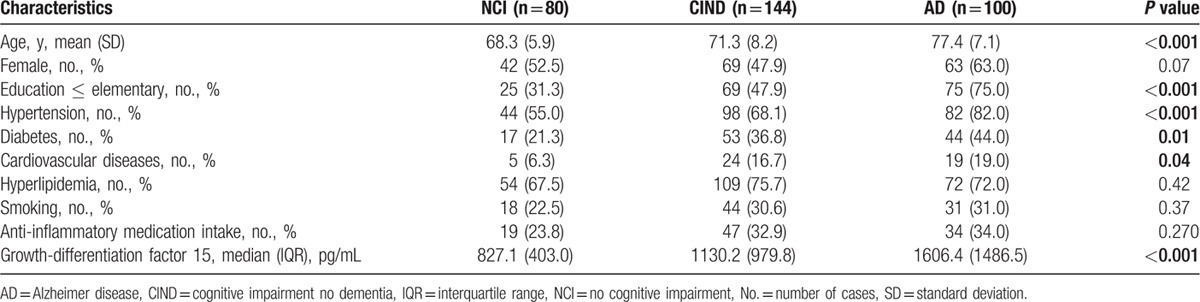
Baseline characteristics of the participants based on their cognitive categories (n = 324).

## 3 Results

A total of 410 subjects were recruited into this study from August 2010 till end July 2014, of which 347 subjects gave consent to have plasma samples available for biomarker assays. Of these, 80 (23.1%) were NCI, 144 (41.5%) were diagnosed with CIND and 100 (28.8%) with AD. Among the 144 subjects with CIND, 70 (48.6%) were classified as having significant CeVD on MRI. For the 100 subjects with AD, 57 (57.0%) had significant CeVD on MRI. The remaining 23 (6.6%) subjects diagnosed with VaD were not included in the present study (see Materials and Methods). Table 1 shows the baseline characteristics of the study subjects. Compared to NCI, subjects with CIND and AD were older, had lower education together with higher incidence of hypertension, diabetes, and cardiovascular disease. For GDF-15 measurements, significantly higher levels were found in both CIND and AD groups compared to NCI (Kruskal–Wallis *P* < 0.001). Furthermore, GDF-15 levels were higher in subjects who took NSAIDs and anti-inflammatories (median [interquartile range] = 1518.1 [1460.3] pg/mL) compared to those who did not (1046.0 [800.7] pg/mL, Mann–Whitney *P* < 0.001), even though the proportions of subjects on anti-inflammatory medications did not differ significantly amongst the diagnostic groups (Table 1). Therefore, we retained anti-inflammatory medication as a covariate in subsequent analyses.

### 3.1 Association of GDF-15 with CIND and dementia in the presence or absence of significant CeVD

Given that a proportion of the subjects from each cognitive group showed significant CeVD on MRI scans, we assessed the potential links between GDF-15 and CeVD using multivariate regression analyses. While higher GDF-15 levels were significantly associated with CeVD among CIND and AD subjects, no association is observed among the NCI subjects, suggesting that the NCI –CeVD and NCI +CeVD subgroups did not differ from each other in terms of GDF-15 levels (Supplementary Table S1). Hence, we proceeded to combine the two NCI subgroups in subsequent analyses. Categorization of GDF-15 into tertiles resulted in two boundary cut-points at 902.7 and 1563.3 pg/mL. Table 2 shows that the highest tertile of GDF-15 was significantly associated with AD after adjustment for covariates (age, education, hypertension, diabetes, anti-inflammatory medication intake, and cardiovascular disease). When stratified by the presence or absence of significant CeVD, Table 3 shows that, even after correcting for covariates, the highest tertiles of GDF-15 were associated with both CIND and AD with significant CeVD, but not with CIND and AD without significant CeVD. Since age is a well-recognized risk factor of cognitive impairment but was not well-matched between the diagnostic groups, we also adjusted the models for age-squared as a conservative approach to account for its confounding effect,^[21]^ and found that associations mentioned earlier remained significant with age-squared adjustment (data not shown).

**Table 2:**
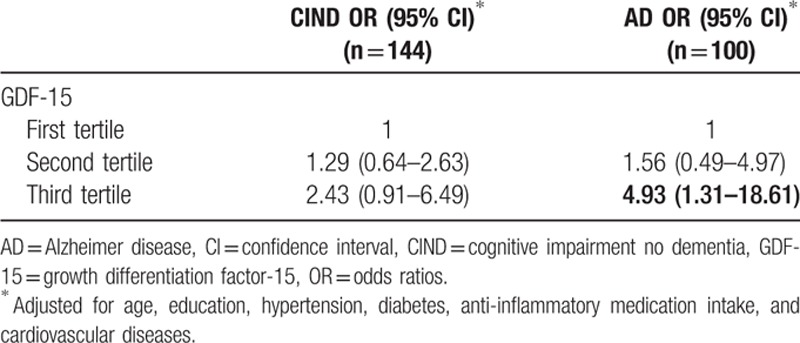
The association between GDF-15 (in tertiles) with CIND and dementia, expressed as odds ratios with 95% confidence intervals.

**Table 3:**
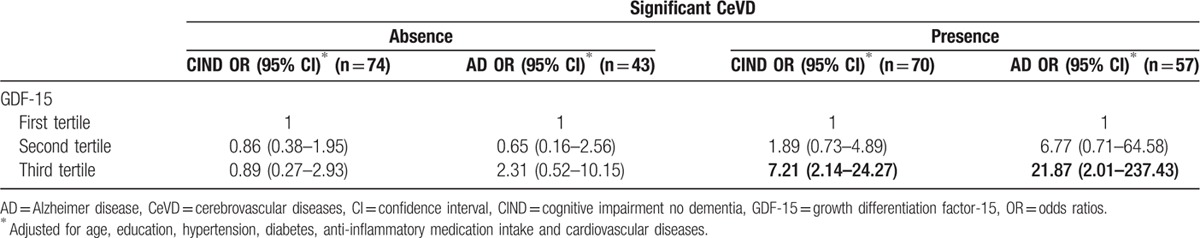
The association between GDF-15 (in tertiles) with CIND and dementia stratified by presence and absence of significant CeVD, expressed as odds ratios with 95% confidence intervals.

### 3.2 Association of GDF-15 with white matter hyperintensities

Table 4 shows the relationship of GDF-15 with CeVD markers on MRI scans, namely WMH (ARWMC scores ≥8), presence of cortical infarct, and presence of 2 lacunes or more. While there was no significant association with cortical infarct and lacunes, higher GDF-15 levels were significantly associated with WMH. Additionally, regression analyses using the same ARWMC criteria (score ≥ 8 vs < 8) to stratify the cognitive groups showed that GDF-15 was significantly associated with both CIND (OR: 13.57, 95%CI: 3.11–59.11) and AD (OR: 14.82, 95%CI: 1.32–166.41) in subjects with ARWMC score ≥8.

**Table 4:**
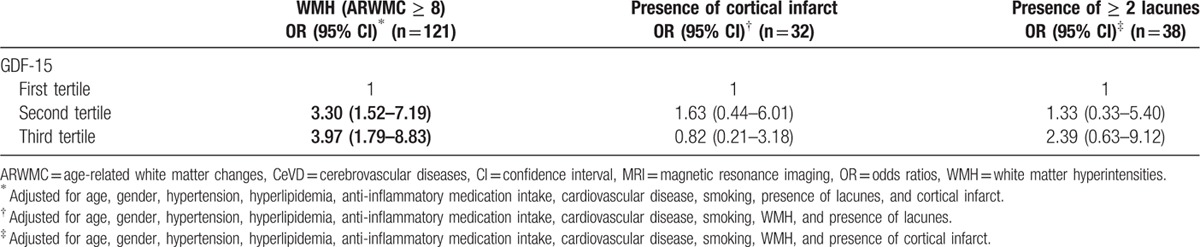
The association between GDF-15 (in tertiles) with MRI markers of CeVD, expressed as odds ratios with 95% confidence intervals.

### 3.3 Sensitivity analyses

Because GDF-15 levels are known to be associated with myocardial infarcts and other cardiovascular diseases,^[14,15]^ we performed sensitivity analyses of their independent associations with CeVD by repeating the multivariate regression analyses after excluding subjects with cardiovascular diseases (total n = 48, including 5 NCI, 24 CIND and 19 dementia, see Table 1). Supplementary Tables S2–S5 denote a repeat of our analyses after exclusion of subjects with cardiovascular diseases, and show that associations between GDF-15 and cognitive impairment, CeVD and in particular WMH remained statistically significant. In fact, Supplementary Table S3 shows that, in the absence of cardiovascular diseases, GDF-15 is associated with both subgroups of cognitive impairment (CIND as well as AD). Taken together, these results suggest that in subjects with cognitive impairments, GDF-15 has an association with CeVD, which is independent of any potential associations with cardiovascular disease.

## 4 Discussion

In this study, we report that higher plasma levels of GDF-15 is associated with CIND and AD only in the presence of significant WMH, independent of other vascular risk factors. To date, this is the only study, which attempted to investigate associations between GDF-15 and neuroimaging findings of CeVD in patients with CIND as well as AD. Our data thus extend the previously reported associations between higher peripheral GDF-15 and cognitive impairment in patients with mild cognitive impairment,^[6]^ and further demonstrate that this association is found across the spectrum of cognitive impairment from CIND to AD. Given the synergistic or additive effects of CeVD on dementia severity in AD,^[3–5]^ our study thus proposes GDF-15 as a potential prognostic biomarker for AD with CeVD. Furthermore, GDF-15 was associated with CeVD independently of its involvement in cardiovascular disease,^[14,15]^ but both associations may be mechanistically analogous. For instance, increased GDF-15 is postulated be a response alongside macrophage activation during ischemic and inflammatory processes arising from atherosclerosis within coronary vessel walls, which is in turn associated with increased cardiovascular risk.^[14,39]^ Therefore, our current data suggest analogous associations between GDF-15 and processes linked to brain ischemia and CeVD.

Interestingly, GDF-15 levels were shown to correlate with WMH, but not with cortical infarcts and lacunes. While in concordance with a previous report on the associations between GDF-15 and WMH in a nondemented cohort,^[19]^ our results added further insights by showing similar GDF-15-WMH association among cognitively impaired as well as demented subjects, independent of various vascular risk factors (Table 4). The mechanisms underlying this are unclear, but white matter structures may be particularly sensitive to hypoxic-ischemic conditions due in part to the selective vulnerability of oligodendrocytes and their progenitors which play critical roles in remyelination and white matter recovery after injury.^[40,41]^ Furthermore, oligodendrocyte progenitors at sites of ischemic injury may also be susceptible to attack by inflammatory, activated macrophages,^[41]^ which also induce GDF-15 expression.^[42]^ Given that white matter integrity is essential for normal brain function including cognition,^[43]^ it is not surprising that we and others have reported WMH association with cognitive impairment in aging and dementia.^[44,45]^ Taken together, the current data therefore suggest that increased peripheral GDF-15 may be a marker for WMH-associated cognitive impairments.

Our study's strengths include the use of comprehensive neuropsychological assessments to diagnose cognitive impairment and dementia as well as the use of 3T-MRI to grade and classify individuals with CeVD. Furthermore, by incorporating multiple covariates in our analytical models, we have taken into account possible confounding effects of demographic characteristics and vascular risk factors. However, several limitations are also apparent. Firstly, the cross-sectional design of this study does not allow examination of the temporal association between GDF-15 and the progression of cognitive impairment, and as cases and majority of the controls were derived from the memory clinic, who may have had a higher burden of CeVD (due to increased prevalence of vascular risk factors), our findings may also be less generalizable to the elderly population at large. Furthermore, there were relatively small numbers of cases with infarct and lacunae; hence, we may be underpowered to detect associations with infarct and/or lacunes. Additionally, potential mechanistic associations between GDF-15 and other putative biomarkers, such as brain natriuretic peptide, cardiac troponin-T, C-reactive protein, and markers of insulin resistance remain to be studied.^[36,46–48]^ Moreover, although we controlled for potential effects of NSAID medications on GDF-15 levels, other concurrently administered medications such as statins and proton-pump inhibitors may need to be considered as well, although in the case of statins, a previous study did not show any associations with GDF-15.^[46]^ Lastly, while we have deliberately excluded VaD subjects in the present study due to our focus on markers of CeVD in AD, epidemiological studies have shown that at least a proportion of CIND with CeVD would go on to develop VaD.^[49,50]^ Therefore, the current data showing higher GDF-15 levels in CIND with CeVD suggest that, besides AD, GDF-15 may also be associated with early stages of VaD, necessitating follow-up studies to elucidate the potential involvement of GDF-15 in VaD.

## 5 Conclusion

The present study finds that GDF-15 is associated with CIND and AD with CeVD, and may have clinical utility as a peripheral biomarker of WMH-associated cognitive impairments. The data also suggest that anti-inflammatory interventions may be a rational therapeutic strategy for cognitive impairment and WMH, given that increased GDF-15 may represent responses toward a heightened inflammatory state in the disease brains. However, further investigations are needed to delineate the precise roles of GDF-15, for example, whether it is produced as an anti-inflammatory response toward cerebrovascular insults, or as part of the pathogenic mechanism accompanying macrophage activation.^[6]^ Follow-up studies on the longitudinal relationship between plasma GDF-15 and cognitive decline for dementia of different etiologies are also needed in order to better assess its clinical prognostic and diagnostic utility.

## Supplementary Material

Supplemental Digital Content
