# Supplementary material for: Growth differentiation factor-15 and white matter hyperintensities in cognitive impairment and dementia
Source: Medicine (Baltimore). 2016 Aug 19;95(33):e4566. doi: 10.1097/MD.0000000000004566 (PMC5370808; doi:10.1097/MD.0000000000004566)
Supplement: Supplemental Digital Content [file medi-95-e4566-s001.doc]

Chai *et al.* Growth Differentiation Factor-15 and White Matter Hyperintensities in Cognitive Impairment and Dementia

**Supplementary Table S1: The association between GDF-15 (in tertiles) with CeVD, among all subjects as well as within each cognitive groups, expressed as odds ratios with 95% confidence intervals**

|  | **Significant cerebrovascular disease (CeVD)** | | | |
| --- | --- | --- | --- | --- |
|  | **In all subjects**  **OR (95% CI)*** | **Among NCI subjects**  **OR (95% CI)*** | **Among CIND subjects**  **OR (95% CI)*** | **Among AD subjects**  **OR (95% CI)*** |
| ***N*** |  |  |  |  |
| Without CeVD | 180 | 63 | 74 | 43 |
| With CeVD | 144 | 17 | 70 | 57 |
| ***GDF-15*** |  |  |  |  |
| 1^st^tertile | 1 | 1 | 1 | 1 |
| 2^nd^tertile | **2.43 (1.22-4.85)** | 1.11 (0.23-5.32) | 2.22 (0.83-5.92) | **14.53 (1.12-188.22)** |
| 3^rd^tertile | **3.62 (1.76-7.43)** | 0.86 (0.10-7.73) | **4.68 (1.65-13.27)** | **12.50 (1.02-152.67)** |

Abbreviations: CeVD, cerebrovascular diseases; NCI, no cognitive impairment; CIND, cognitive impairment no dementia; AD, Alzheimer’s disease; OR, odds ratios; CI, confidence interval; N, number of cases.

*Adjusted for age, hypertension, anti-inflammatory medication,cardiovascular disease and smoking.

Chai *et al.* Growth Differentiation Factor-15 and White Matter Hyperintensities in Cognitive Impairment and Dementia

**Supplementary Table S2: Baseline characteristics of the participants based on their cognitive categories (n=276) excluding subjects with cardiovascular diseases (n = 48)**

| **Characteristics** | **NCI**  **(n=75)** | **CIND**  **(n=120)** | **AD**  **(n=81)** | **P value** |
| --- | --- | --- | --- | --- |
| Age, years, mean (SD) | 68.3 (6.1) | 71.0 (7.8) | 77.2 (7.3) | **<0.001** |
| Female, no. (%) | 41 (54.7) | 62 (51.7) | 54 (66.7) | 0.098 |
| Education ≤ elementary, no. (%) | 21 (14.8) | 59 (49.2) | 62 (76.5) | **<0.001** |
| Hypertension, no. (%) | 40 (53.3) | 76 (63.3) | 65 (80.2) | **0.002** |
| Diabetes, no. (%) | 16 (21.3) | 42 (35.0) | 35 (43.2) | **0.014** |
| Hyperlipidemia, no. (%) | 49 (25.7) | 87 (72.5) | 55 (67.9) | 0.548 |
| Smoking, no. (%) | 16 (21.3) | 28 (23.3) | 25 (30.9) | 0.333 |
| Anti-inflammatory medication intake, no. (%) | 15 (20.0) | 37 (31.1) | 21 (25.9) | 0.232 |
| Growth-differentiation factor 15, median (IQR), pg/ml | 812.8 (325.6) | 1046.7 (936.7) | 1598.7 (1566.1) | **<0.001** |

NCI = no cognitive impairment, CIND = cognitive impairment no dementia, SD = standard deviation, No. = number of cases, IQR = interquartile range.

Chai *et al.* Growth Differentiation Factor-15 and White Matter Hyperintensities in Cognitive Impairment and Dementia

**Supplementary Table S3: The association between GDF-15 (in tertiles) with CIND and dementia, expressed as odds ratios with 95% confidence intervals, excluding subjects with cardiovascular diseases (n = 48)**

|  | **CIND**  **OR (95% CI)***  **(n= 144)** | **AD**  **OR (95% CI)***  **(n= 100)** |
| --- | --- | --- |
| *GDF-15* |  |  |
| 1^st^tertile | 1 | 1 |
| 2^nd^tertile | 1.32 (0.63-2.76) | 1.22 (0.37-4.04) |
| 3^rd^tertile | **4.15 (1.39-12.41)** | **5.41 (1.29-22.76)** |

GDF-15 = growth differentiation factor-15, CIND = cognitive impairment no dementia, OR = odds ratios, CI = confidence interval.

* Adjusted for age, education, hypertension, diabetes and anti-inflammatory medication.

Chai *et al.* Growth Differentiation Factor-15 and White Matter Hyperintensities in Cognitive Impairment and Dementia

**Supplementary Table S4: The association between GDF-15 (in tertiles) with CIND and dementia stratified by presence and absence of significant CeVD, expressed as odds ratios with 95% confidence intervals, excluding subjects with cardiovascular diseases (n = 48)**

|  | **Significant CeVD** | | | |  |
| --- | --- | --- | --- | --- | --- |
|  | **Absence** | | **Presence** | |  |
|  | **CIND**  **OR (95% CI)***  **(n=74)** | **AD**  **OR (95% CI)***  **(n=43)** | **CIND**  **OR (95% CI)***  **(n=70)** | **AD**  **OR (95% CI)***  **(n=57)** | |
| *GDF-15*  1^st^tertile | 1 | 1 | 1 | 1 | |
| 2^nd^tertile | 0.86 (0.37-2.01) | 0.52 (0.12-2.18) | 2.56 (0.90-7.27) | 5.65 (0.57-56.32) | |
| 3^rd^tertile | 1.64 (0.47-5.77) | 2.37 (0.49-11.56) | **15.60 (3.62-67.22)** | **27.35 (2.20-339.47)** | |

GDF-15 = growth differentiation factor-15, CeVD = cerebrovascular diseases, CIND = cognitive impairment no dementia, AD = Alzheimer’s disease, OR = odds ratios, CI = confidence interval.

* Adjusted for age, education, hypertension, diabetes and anti-inflammatory medication.

Chai *et al.* Growth Differentiation Factor-15 and White Matter Hyperintensities in Cognitive Impairment and Dementia

**Supplementary Table S5: The association between GDF-15 (in tertiles) with MRI markers of CeVD, expressed as odds ratios with 95% confidence intervals, excluding subjects with cardiovascular diseases (n = 48)**

|  | **WMH (ARWMC≥8)**  **OR (95% CI)***  **(n=121)** | **Presence of cortical infarct**  **OR (95% CI)†**  **(n=32)** | **Presence of ≥ 2 lacunes**  **OR (95% CI)‡**  **(n=38)** |
| --- | --- | --- | --- |
| *GDF-15* |  |  |  |
| 1^st^tertile | 1 | 1 | 1 |
| 2^nd^tertile | **3.69 (1.57-8.67)** | 2.59 (0.42-16.14) | 1.18 (0.23-5.97) |
| 3^rd^tertile | **4.94 (2.02-12.07)** | 1.09 (0.16-7.39) | 2.33 (0.49-11.15) |

MRI = magnetic resonance imaging, CeVD = cerebrovascular diseases, WMH = white matter hyperintensities, ARWMC = age related white matter changes, OR = odds ratios, CI = confidence interval

* Adjusted for age, gender, hypertension, hyperlipidemia, smoking, anti-inflammatory medication, presence of lacunes and cortical infarct.

† Adjusted for age, gender, hypertension, hyperlipidemia, smoking, anti-inflammatory medication, WMH and presence of lacunes.

‡ Adjusted for age, gender, hypertension, hyperlipidemia, smoking, anti-inflammatory medication, WMH and presence of cortical infarct.
